# Supplementary figures and images for: Navigating the Intersection of Glycemic Control and Fertility: A Network Perspective
Source: Int J Mol Sci. 2024 Sep 16;25(18):9967. doi: 10.3390/ijms25189967 (PMC11432572; doi:10.3390/ijms25189967)

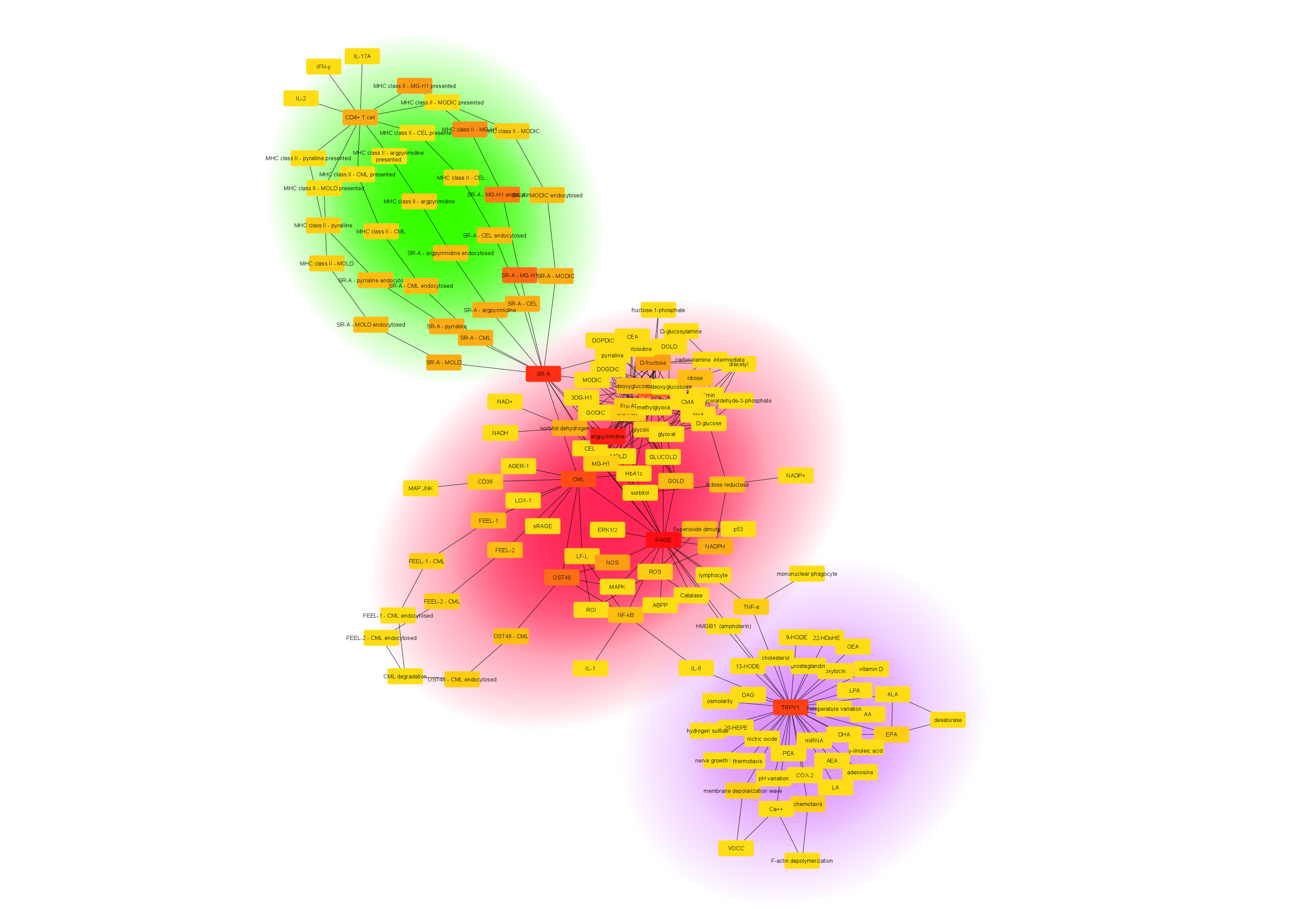

Supplement: Supplementary file 1 [file ijms-25-09967-s001.zip › Supplementary 3.jpeg]
